# Imaging Ca^2+^ activity in mammalian cells and zebrafish with a novel red-emitting aequorin variant

**DOI:** 10.1007/s00424-014-1639-3

**Published:** 2014-10-31

**Authors:** Adil Bakayan, Beatriz Domingo, Atsushi Miyawaki, Juan Llopis

**Affiliations:** 1 Centro Regional de Investigaciones Biomédicas (CRIB) and Facultad de Medicina de Albacete, Universidad Castilla-La Mancha, C/ Almansa 14, 02008 Albacete, Spain; 2 Present Address: Campus CNRS-Gif, INAF/UPR3294 - N&D, 1 Avenue de la Terrasse, Bat 32-33, 91198 Gif sur Yvette Cedex, France; 3 Laboratory for Cell Function Dynamics, Brain Science Institute, RIKEN, Wako-city, Saitama 351-0198 Japan

**Keywords:** Bioluminescence, Resonance energy transfer, Calcium, Aequorin, Coelenterazine, Red fluorescent protein

## Abstract

Ca^2+^ monitoring with aequorin is an established bioluminescence technique, whereby the photoprotein emits blue light when it binds to Ca^2+^. However, aequorin’s blue emission and low quantum yield limit its application for in vivo imaging because blue-green light is greatly attenuated in animal tissues. In earlier work, aequorin was molecularly fused with green, yellow, and red fluorescent proteins, producing an emission shift through bioluminescence resonance energy transfer (BRET). We have previously shown that the chimera tandem dimer Tomato-aequorin (tdTA) emits red light in mammalian cells and across the skin and other tissues of mice [1]. In this work, we varied the configuration of the linker in tdTA to maximize energy transfer. One variant, named Redquorin, improved BRET from aequorin to tdTomato to almost a maximum value, and the emission above 575 nm exceeded 73 % of total counts. By pairing Redquorin with appropriate synthetic coelenterazines, agonist-induced and spontaneous Ca^2+^ oscillations in single HEK-293 cells were imaged. In addition, we also imaged Ca^2+^ transients associated with twitching behavior in developing zebrafish embryos expressing Redquorin during the segmentation period. Furthermore, the emission profile of Redquorin resulted in significant luminescence crossing a blood sample, a highly absorbing tissue. This new tool will facilitate in vivo imaging of Ca^2+^ from deep tissues of animals.

**Electronic supplementary material:**

The online version of this article (doi:10.1007/s00424-014-1639-3) contains supplementary material, which is available to authorized users.

## Introduction

Ca^2+^ acts as a second messenger in the control of a myriad of cellular processes. Among all Ca^2+^ sensors available, aequorin (Aeq) is a reporter of choice in numerous applications owing to its remarkable flexibility. It is a Ca^2+^-sensitive photoprotein, a complex of the protein apoaequorin, the luminophore coelenterazine (CLZ), and molecular oxygen. Aeq luminescence has a maximum from 450 to 470 nm, depending on the CLZ. Its functional properties have been altered for various purposes by using synthetic CLZ analogs [27]. In addition, mutagenesis has provided variants with different Ca^2+^ sensitivity, emission shifts, improved thermostability, and other features [4, 12, 19, 30, 32]; mutant Aeq and synthetic CLZs can also be combined [8, 24].

An important but elusive goal has been to shift Aeq’s blue emission to red. This is a key property to allow Ca^2+^ measurements in deep tissues of living animals. Because of the nature of light absorption and scattering by tissues, to be maximally transmitted, a probe should provide strong emission in the optical window for intravital imaging, from 600 to 900 nm [14, 34].

Bioluminescence resonance energy transfer (BRET) between Aeq (energy donor) and a fluorescent protein (FP) molecularly fused to it (energy acceptor) has been used to shift Aeq emission and generate green, yellow, and red hues [1, 2, 7, 15]. The first red FP fused with Aeq was mRFP, which has an emission peak at 612 nm [5], but energy transfer was rather limited [7, 15]. More recently, we used tdTomato (582-nm emission maximum) [26] as a BRET acceptor. The chimera tandem dimer Tomato-Aeq (tdTA) showed the highest percentage of counts above 600 nm of all previous fusions, but energy transfer did not exceed 50 % [1]. In the present work, we modified tdTA to obtain a bright red bioluminescent Ca^2+^ sensor. To improve BRET, the distance between tdTomato and Aeq was decreased and a point mutation was introduced in Aeq. The best functional variant was combined with appropriate CLZ analogs and tested for its ability to image Ca^2+^ upon agonist stimulation, as well as spontaneous Ca^2+^ activity in single mammalian cells. In addition, in zebrafish embryos, microinjection of messenger RNA (mRNA) coding for the chimera and its subsequent expression allowed monitoring Ca^2+^ transients during early development.

## Materials and methods

### Protein engineering

**Fig. 1:**
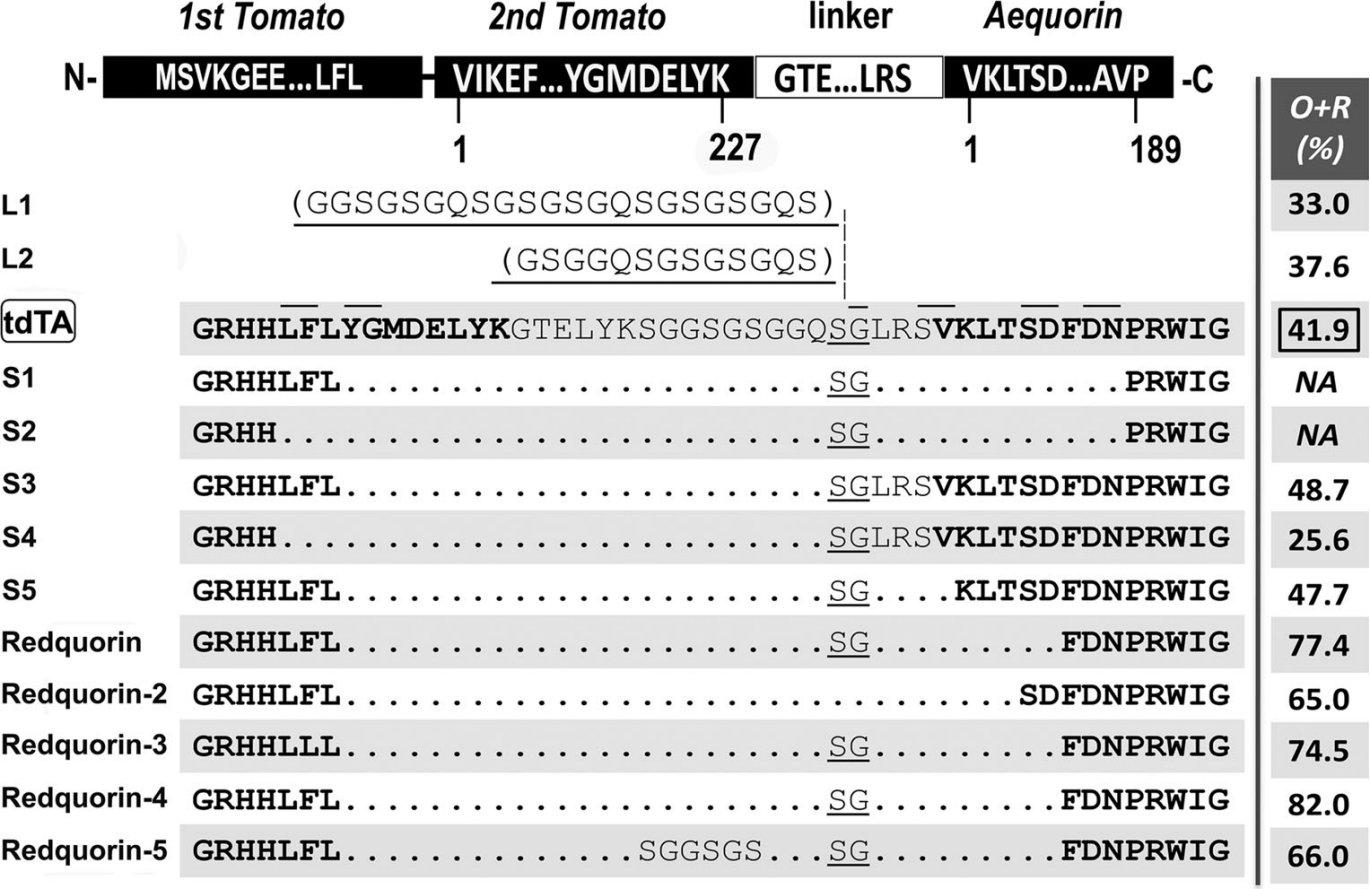
tdTomato-Aequorin (tdTA) variants with modified linker and deletions in tdTomato or Aeq. A scheme of the starting chimera tdTA is shown on *top* (amino acids in one-letter code). The modifications affected the C-t of tdTomato, the linker, and the N-t of Aeq. The underlined dipeptide linker SG was conserved in most variants and contained insertions in variants L1 and L2 (*L* stands for long and *S* for short variants). *Horizontal lines* above tdTA sequence indicate mutagenic sites for generating different variants. The *rightmost column* shows the percentage of light of each variant into the 595- and 640-nm emission channels (O + R, *orange* plus *red*), measured as indicated in the Materials and methods section. Redquorin-4 and Redquorin-5 contain the Aeq point mutation Y82F (not shown on the sequence)

Variants with different linkers between tdTomato and Aeq were constructed based on tdTA in pCDNA3 (Invitrogen), developed in our previous work [1]. tdTA comprised tdTomato, a 20-amino acid linker and Aeq (Fig. 1). The short linker variants of tdTA (S1, S2, S3, S4, S5, and Redquorin) were obtained by site-directed mutagenesis, converting the sequence coding for some amino acid pairs (marked by a line above tdTA in Fig. 1) into a *Kpn*2I site, followed by digestion and religation to yield the different clones. The mutagenic oligonucleotides G-D, F224L, and Y82F (Supplementary Table 2) were applied on Redquorin to generate variants Redquorin-2, Redquorin-3, and Redquorin-4, respectively. To construct the long linker variants, a unique *Kpn*2I restriction site coding for dipeptide SG (underlined in Fig. 1) was placed between the linker and Aeq in tdTA. Complementary oligonucleotides 22aa-L, 13aa-L, and 6aa-L (Supplementary Table 2) were annealed; the resulting double-stranded DNA with overhang extensions were inserted into the *Kpn*2I site of tdTA and Redquorin-4 to yield variants L1, L2, and Redquorin-5, respectively. The bacterial expression vectors coding for CitA, tdTA, and Redquorin were constructed by digestion of pcDNA3 clones with *Hind*III and *Xho*I and subcloning into pTriEX-4 (Novagen) [1]. Restriction enzymes and T4 DNA ligase were from Fermentas, and multi-site mutagenesis kit was from Stratagene. All clones were verified by DNA sequencing.

### Cell culture

HEK-293 cells were cultured in minimum essential medium (MEM)-alpha supplemented with 4 mM l-glutamine. HeLa cells (American Type Culture Collection) were cultured in DMEM supplemented with 2 mM l-glutamine. Both media were supplemented with 10 % heat-inactivated fetal bovine serum (FBS), penicillin (100 U/mL), and streptomycin (100 μg/mL) (tissue culture reagents were from Lonza). Cells were maintained in a humid atmosphere at 37 °C with 5 % CO_2_. Cells were seeded at a density of 6 × 10^5^ cells/cm^2^ and transfected 1 day after seeding with Lipofectamine 2000 (Invitrogen) according to the manufacturer’s instructions. For imaging experiments, cells were seeded onto glass-bottom dishes (35 mm, Ibidi).

### Live cell bioluminescence experiments

#### Aeq reconstitution

Cells expressing the chimeric proteins were washed twice with Hank’s Balanced Salt Solution (HBSS) and incubated in the dark with 3 μM of the desired CLZ in OptiMEM I medium (Gibco) supplemented with 1 % FBS for 2–3 h at 37 °C and 5 % CO_2_. Cells were washed twice and maintained in HBSS for imaging. CLZ-*native*, CLZ-*f*, and CLZ-*hcp* were from Biotium, and CLZ-*h* was from Invitrogen.

#### Ca^2+^ imaging in live mammalian cells

Bioluminescence imaging was performed in the dark in a microscopy setup as previously described [1]. Light emission from cells was collected with an oil-immersion objective into emission filters held in a filterwheel (or no filter, as indicated), and captured by an EM-CCD camera (EMC9100-13, Hamamatsu Photonics).

#### Spectral characterization in live mammalian cells

The emission spectrum of variants was evaluated in single live HeLa or HEK cells by using four emission channels, as described previously [1]. Briefly, the percent contribution of Ca^2+^-elicited bioluminescence was quantified using four bandpass emission filters (481/34, 535/52, 595/40, and 640/50 nm, center wavelength/bandwidth; B, G, O, and R filters, respectively). All counts were corrected for background and normalized for filter bandwidth, for transmittance, and for the nominal spectral sensitivity of the EM-CCD camera.

#### Sensitivity of the variants to Ca^2+^ in single cells

HEK cells were transiently transfected (24 h) with different FP-Aeq variants, incubated with CLZ, and placed on the microscope stage. A solution of carbachol (50 μM) (Sigma) was perfused, and bioluminescence was recorded. Cells were permeabilized with digitonin (30 μM) (Sigma) to allow saturation of Aeq with Ca^2+^ (1.3 mM extracellular), and total light emission was integrated. Then, we calculated the fractional rate of photoprotein consumption (*L*/*Lmax*), which is the light emitted at each time point (*L*, per second) divided by total remaining luminescence (*Lmax*, the integral of light emission from that point until full exhaustion of the photoprotein). Digitonin used to discharge all Aeq counts from single cells did not cause the immediate release of tdTA or Redquorin into the medium, thus avoiding potential underestimation of *Lmax*. By the time all bioluminescence was emitted, most of tdTA/Redquorin was still trapped in the cytoplasm, as judged by fluorescence. The *L*/*Lmax* values obtained during perfusion with carbachol provided an estimate of Ca^2+^ sensitivity in live cells.

### In vitro characterization

#### Cell extracts for spectroscopic characterization

One day after transfection, HeLa cells expressing the chimeric proteins were scraped in cold phosphate-buffered saline (PBS), harvested at 500×*g* for 5 min, resuspended in cold buffer (10 mM dithiothreitol, 5 mM ethylene glycol-bis(2-aminoethylether)-N,N,N′,N′-tetraacetic acid (EGTA), and 5 μM CLZ-*f* in PBS) and incubated for 3 h at 4 °C in the dark to reconstitute Aeq. Subsequently, cells were washed with PBS and lysed with a hypoosmotic buffer containing 20 mM Tris–HCl (pH 7.5), 10 mM EGTA, 5 mM dithiothreitol, and a protease inhibitor cocktail (complete-Mini, EDTA-Free, Roche). The cell membranes were broken by two freeze-thaw cycles, followed by passage through a 25-g needle (four times). The resulting lysates were centrifuged at 13,000×*g* for 1 min at 4 °C. Supernatants containing the active FP-Aeq were recovered and stored at −20 °C for later use.

#### Protein expression in *E. coli* and purification

Expression of His-tagged recombinant hybrid proteins (tdTA, Redquorin, and CitA) in *E. coli* was carried out using pTriEx-4 plasmid as described previously [1] with a few modifications. Following sonication, the efficiency of protein extraction was increased by using five freeze-thaw cycles and cell lysates were centrifuged to eliminate debris. His-tagged proteins in the supernatants were purified in columns with Ni-NTA HisBind Resin (Novagen) according to the manufacturer’s protocol. Buffer was exchanged using molecular weight cutoff filters of 30 kDa (CitA) or 50 kDa (tdTA and Redquorin) (AmiconUltra, Millipore). One milliliter of protein sample was concentrated into 50 μL and resuspended in 1 mL of the desired buffer; this step was repeated three to four times. Protein content was quantified with a spectrophotometer (Nanodrop) by absorbance at 280 nm or at the wavelength corresponding to the absorbance peak of the FP fluorochrome in the chimera (516 nm for CitA; 554 nm for tdTA and Redquorin). Purified samples were stored at 4 °C.

#### Native gel electrophoresis

Integrity of the purified protein samples was assayed in a non-denaturing 6 % polyacrylamide gel. The native gel was visualized by fluorescence. Citrine-containing bands were revealed by setting the monochromator (C7773, Hamamatsu) at 490 nm, whereas tdTomato was excited at 540 nm. Emission filters (yellow for CitA; red for tdTA and Redquorin) were placed in front of the objective of a digital camera (DSC-S500, Sony) to acquire the images. Then, the gel was Coomassie-stained to reveal all protein bands (imaged with FujiFILM LAS-3000).

#### Aeq reconstitution of purified samples

Aeq samples were buffer-exchanged to 50 mM Tris–HCl and 150 mM NaCl (pH 7.5). Aeq was reconstituted in vitro by adding 5 μM CLZ-*f* or CLZ-*native*, 10 mM dithiothreitol, and 5 mM EGTA, and incubated overnight at 4 °C, in the dark [1].

#### Spectral analysis

Spectral measurements of tdTA variants were performed in the laboratory of Dr. Philippe Brûlet (CNRS, Gif-sur-Yvette, France). An aliquot of purified protein samples or cell lysates was brought in contact with a saturating Ca^2+^ solution (50 mM Tris–HCl, 50 mM CaCl_2_, pH 7.5), in a PCR tube to induce light emission. Emitted photons were collected by an optic fiber, guided into a spectrometer (Specim), and captured by an EM-CCD camera (Andor DU-897 back illuminated). Spectral calibration was done with laser pointers (405 and 650 nm). This setup allowed immediate and synchronous spectral analysis (no scanning) of emitted luminescence at 1-nm resolution [7].

#### Ca^2+^ sensitivity curves

Purified samples reconstituted with CLZ were washed and buffer-exchanged to zero Ca^2+^ buffer (10 mM 3-morpholinopropane-1-sulfonic acid (MOPS), 100 mM KCl, 1 mM K_2_EGTA, pH 7.2). A 10-μl aliquot of protein sample (30–50 ng/μl) was placed in a luminometer (Sirius, Berthold Detection Systems); luminescence emission was measured for 20 s (background); and 400 μl of a solution containing the desired concentration of free Ca^2+^ (EGTA-buffered solutions from Molecular Probes) were added. Acquisition continued until a stable or decaying signal was obtained. At this point, 500 μl of a saturating Ca^2+^ solution (10 mM MOPS, 50 mM CaCl_2_, pH 7.2) were injected to consume Aeq and light was integrated until intensity returned to background level. Luminescence intensity (*L*) at different [Ca^2+^] was taken as the response peak value, in case of decaying signal, or as the value of stable emission. Luminescence maximum (*Lmax*) was measured by integrating all remaining counts. The EC_50_ values were extracted from a sigmoidal dose–response fit using Prism4 (GraphPad).

#### Bioluminescence spectra of CitA and Redquorin in blood

To measure the degree of transmission of luminescence of the chimeras in a blood sample, 5 μl of recombinant purified CitA and Redquorin, previously reconstituted with CLZ-*f* were mixed with 30 μl of either buffer (50 mM Tris–HCl, pH 7.5) or EDTA-treated rabbit blood. Using the same setup and protocol as in the section “Spectral analysis,” these mixtures were induced to release light by injection of 100 μl of a high Ca^2+^ solution (resulting in a 4.5-fold final blood dilution) and luminescence spectra were obtained. The spectra were normalized per picomole of each chimera. To determine whether the spectra obtained in blood could be explained by hemoglobin transmittance, the spectra obtained in buffer were multiplied by the wavelength-dependent transmittance of oxyhemoglobin, considering the hemoglobin concentration (33 g/L) and an approximate path length. The hemoglobin transmittance was calculated from the tabulated molar extinction coefficient for hemoglobin in water compiled by Dr. Scott Prahl [13].

#### Western blot analysis

Transfected HeLa cells were lysed and processed as indicated in “Cell extracts for spectroscopic characterization” section without the Aeq reconstitution step. Protein content of the supernatant was estimated by BCA protein assay (Thermo Scientific Pierce). Proteins in the sample (40 μg) were separated by SDS-PAGE (10 %), and bands were transferred onto a Hybond-P membrane (Amersham Biosciences). After blocking with 3 % BSA, the membranes were incubated overnight at 4 °C with mouse anti-GFP (1:2,000; Covance) or mouse anti-DsRed (1:500; BD Pharmingen). Membranes were washed and probed with a sheep anti-mouse IgG HRP conjugate (1:1,000; Santa Cruz) at room temperature for 45 min. SuperSignal West Dura (Thermo Scientific Pierce) was used as chemiluminescence substrate, and detection was carried out with a FujiFILM LAS-3000.

### Bioluminescence imaging in zebrafish embryos

Redquorin from vector pTriEx4-Redquorin was subcloned into *Eco*RI/*Xho*I sites of pCS2+ vector for in vitro mRNA synthesis with mMessage mMachine SP6 kit (Ambion). Redquorin mRNA was microinjected into fertilized one-cell stage zebrafish eggs maintained at 28 °C. Injected embryos were dechorionated using pronase (0.5 mg/mL) and forceps. Fluorescence images in Fig. 6b were acquired with an AxioCam MRc color camera (Zeiss) in a Leica MZ16F stereomicroscope using a red filter cube (BP546/12; 560 beamsplitter; BP605/75). Embryos were anesthetized (Tricaine 0.016 %) for prim-16 and long-pec stage images. For standard bioluminescence experiments, dechorionated embryos from 2 to 3 h post-fertilization (hpf) were incubated with 50 μM of CLZ-*f* or CLZ-*hcp* (Biotium) in E3 medium (5 mM NaCl, 0.17 mM KCl, 0.4 mM CaCl_2_, and 0.16 mM MgSO_4_). For bioluminescence imaging, embryos were immobilized in a 0.3 % agarose chamber on a glass-bottom dish and CLZ (20 μM) was maintained during recording. Time-lapse images were acquired in a light-tight microscope (LV200, Olympus), with a ×20 oil-immersion objective (N.A. 0.85), and an EM-CCD camera (CascadeII:512, Photometrics) controlled by MetaMorph software (Molecular Devices). One brightfield image was acquired every 60 bioluminescence images. Recording of the tail contractions shown in Fig. 6d was done in an embryo with the head embeded in 0.3 % agar and a free tail, submerged in E3 medium. Camera was set to slow readout mode or to stream mode (20 fps; 10 MHz readout) for imaging contractions (Fig. 6d). Image analysis was performed with ImageJ (NIH).

Zebrafish (*Danio rerio*) were maintained in the Center for Zebrafish Research at Okazaki Institute for Integrative Bioscience (RIKEN, Saitama, Japan) according to standard procedures. Embryo staging was done in base of hours after fertilization and morphological criteria.

## Results

### New tandem dimer Tomato-aequorin variants with red shifted emission: linker optimization

We introduced variability in the 20-residue linker between tdTomato and Aeq in the chimera tdTA by deleting or inserting amino acid residues and studied whether these changes affected energy transfer (Fig. 1). Variants L1 and L2 contained insertions in the original tdTA linker, and the remaining chimeras were deletions. In addition, the C terminus of tdTomato was shortened up to 11 residues and some N-terminal deletions on Aeq were tested. Various combinations of the above were constructed and expressed in HeLa cells to examine cell viability and fluorescence. All variants showed diffuse fluorescent staining in transfected cells (tdTomato fluorescence), except for S2 and S5, in which fluorescent aggregates were observed (Supplementary Fig. 1). In addition, all chimeras except for S1 and S2 emitted light when digitonin was added to cells to raise cytoplasmic Ca^2+^.

Bioluminescent emission of the chimeras was characterized in mammalian cells with the four-filter approach that we previously employed [1]. Briefly, during a Ca^2+^ rise induced by digitonin, four emission filters (blue, green, orange, and red) were alternated and the relative counts were calculated (counts in filter × 100 / sum of counts in the four filters). Figure 1 shows the percentage of counts in the orange plus red (O + R) filters as an indication of energy transfer (supplementary Table 1 shows the results in detail).

Increasing the linker size (variants L1 and L2) resulted in blue-shifted chimeras (33 and 38 % of counts in O + R filters, respectively), compared with the starting construct tdTA (Fig. 1). In contrast, deletion of most of the linker plus eight residues from the C terminus of tdTomato (variant S3) improved red emission. Further deletion of three residues of tdTomato (variant S4) had the opposite effect. Thus, as it happens in many protein fusions formed by donor and acceptor FP [9, 29], shorter linkers resulted in more energy transfer from Aeq to tdTomato, but the C terminus of the FP did not tolerate large deletions.

Starting with variant S3, deletion of residues LRS**V** upstream of Aeq (Aeq residues are indicated in bold) had no effect (variant S5), but removal of LRS**VKLTSD** resulted in an outstanding red shift (77 % of collected light was in the O + R filters) (Fig. 1). Because of its red emission and useful functional properties, we will refer to this variant as *Redquorin*. Other changes (variant Redquorin-2: mutation of a Gly in the linker into Asp, which restores Ser-Asp sequence of Aeq N-terminus; variant Redquorin-3: mutation of Phe into Leu at the C-terminus of tdTomato) also red-shifted the emission compared to tdTA but were not better than Redquorin (Fig. 1).

**Fig. 2:**
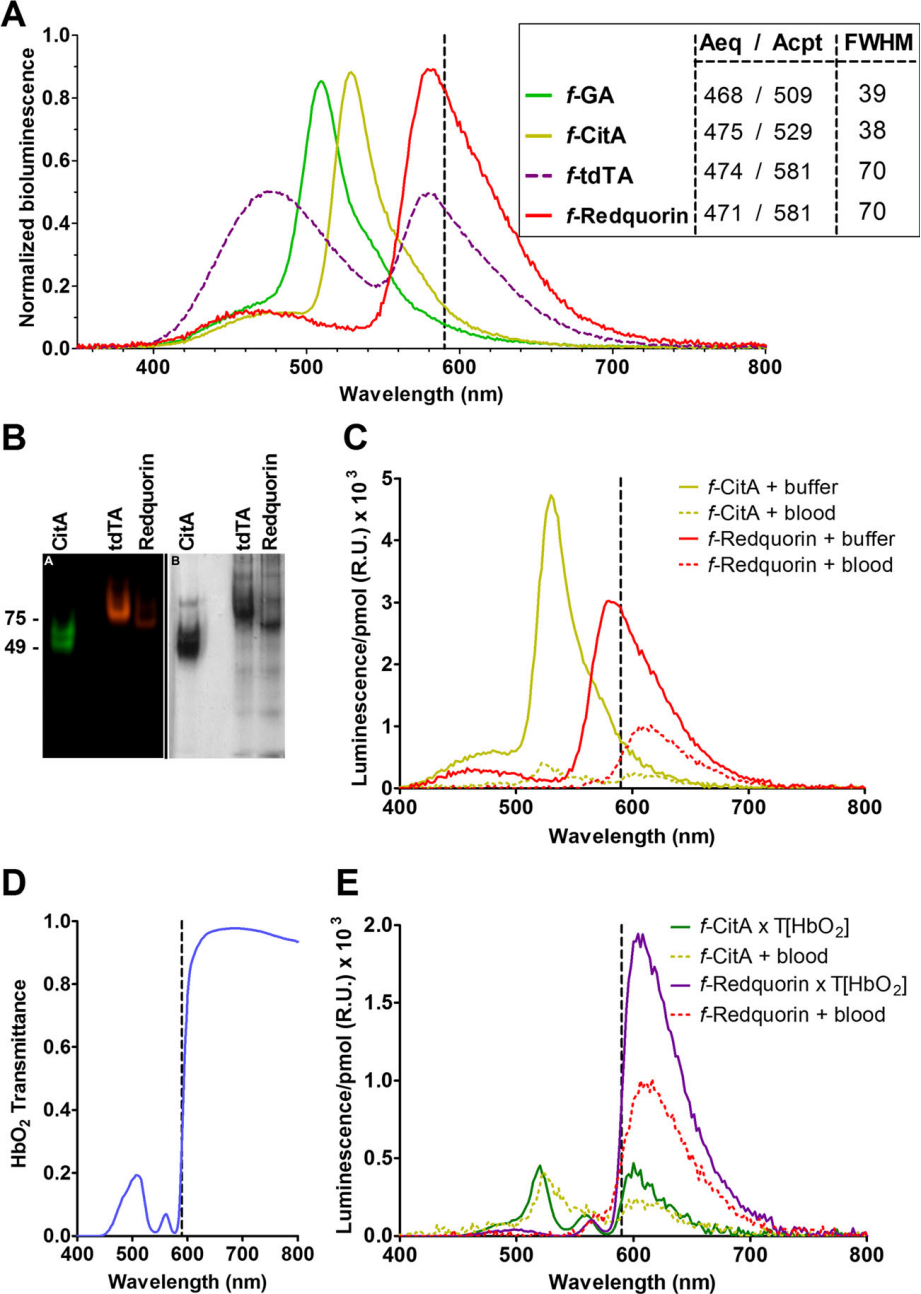
In vitro characterization of tdTA variants. **a** Ca^2+^-triggered bioluminescence spectra of fluorescent protein (*FP*)-Aeq chimeras. The hybrid proteins, obtained from HeLa cell lysates (*GA*) or produced in *E. coli* and affinity column-purified (CitA, tdTA, Redquorin) were reconstituted with CLZ-*f*. The sum of the height of the two peaks (Aeq and fluorescent protein) was normalized to one. The *inset* indicates the wavelength of Aeq (*donor*) and FP (*acceptor*) emission peaks and the full width at half-maximum (*FWHM*) of the FP peak, all expressed in nanometers. **b** The integrity of affinity-purified hybrid proteins CitA, tdTA, and Redquorin was tested on a native gel and visualized by fluorescence (*left*), followed by Coomassie staining (*right*). **c** Bioluminescence spectra per picomole of *f*-CitA and *f*-Redquorin suspended in buffer or blood (blood dilution after addition of Ca^2+^ was 4.5-fold). **d** Transmittance spectrum of a oxyhemoglobin solution. **e** Comparison of *f*-CitA and *f*-Redquorin spectra obtained experimentally in blood (*dotted lines*) with the calculated spectra considering the transmittance of oxyhemoglobin (*full lines*). The *dashed vertical lines* are shown to highlight the luminescence above 590 nm

We obtained the Ca^2+^-dependent luminescence spectrum of affinity-purified Redquorin (Fig. 2a) and earlier FP-Aeq fusions. The integrity of the samples was verified by fluorescence on a native gel (Fig. 2b). The 582-nm peak of Redquorin, due to BRET, was much larger than the corresponding peak of tdTA, and there was an Aeq residual peak at 470 nm. In addition, since the main peak of Redquorin was wider than that of GA or CitA (full width at half-maximum of 70 nm, compared to 38 nm) (Fig. 2a, inset), there was a significant amount of counts above 590 nm, in the optical window for intravital imaging in mammals. Western blots of cytosolic extracts of HeLa cells transfected with tdTA and Redquorin showed unique bands of the expected molecular mass (Supplementary Fig. 2), suggesting that the chimeric proteins were stable within cells.

### Improving red shift with Y82F point mutation

Obelin is a Ca^2+^-activated luminescent photoprotein from marine organisms of the genus *Obelia*, which is red-shifted compared to Aeq. Stepanyuk et al. discovered that obelin and Aeq bioluminescent spectra could be interchanged by substitution of a single active site residue of each photoprotein [30]. Thus, mutation of Aeq Tyr82 into Phe (Y82F) caused a 29-nm red shift. We reasoned that introducing Aeq Y82F mutation in tdTA or its variants could potentially improve BRET by increasing the overlap integral between Aeq luminescence and tdTomato absorption. Furthermore, because this mechanism is independent of the distance considerations described above, it could have an additive effect and further enhance red emission.

In fact, Aeq mutation Y82F on variant S3 (S3-Y82F) resulted in 10 % increase in light collected in O + R filters (from 49 to 59 %, Supplementary Table 1). When it was introduced on Redquorin, 82 % of the counts were collected in O + R filters (see Redquorin-4 in Fig. 1 and Supplementary Table 1). Although Redquorin-4 was the chimera with most counts in the red described in this study, overall, it performed worse than Redquorin in terms of total emitted counts in cells; thus, Redquorin was used in subsequent experiments.

### Redquorin and CitA emission in blood

Animal tissues absorb and scatter blue and green light more than red light. Out of several components responsible for this (like melanin of pigmented tissues), a key factor is hemoglobin present in blood. We determined the Ca^2+^-triggered luminescence spectra of affinity-purified recombinant *f*-CitA and *f*-Redquorin suspended in rabbit blood or in buffer. Light was strongly attenuated when the chimeras were mixed with blood (Fig. 2c), but Redquorin provided significantly more counts than CitA (spectra were normalized per picomole protein). In addition, more than 90 % of Redquorin counts in blood were above 590 nm, proving the point that tissues are quite transparent to red light.

Furthermore, the attenuation and shape of the spectra in blood could be explained. Multiplication of the spectra obtained in buffer (Fig. 2c) by the wavelength-dependent transmittance of oxyhemoglobin (Fig. 2d) resulted in spectra which mimicked that obtained experimentally in blood (Fig. 2e). In conclusion, Redquorin results in stronger luminescence across an animal tissue (such as blood) than green/yellow FP-Aeq chimeras.

### Ca^2+^ sensitivity of tdTA variants in vitro and in live cells

**Fig. 3:**
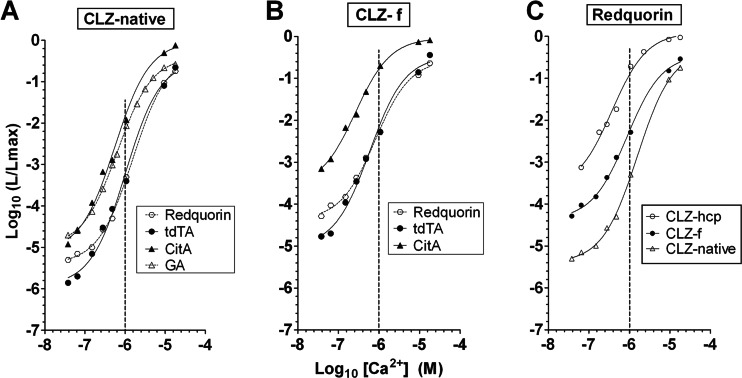
Ca^2+^ calibration curves of recombinant proteins expressed in *E. coli* and affinity purified. The relationship between [Ca^2+^] and (*L*/*Lmax*) is displayed on a log–log plot. **a** Reconstitution with CLZ-*native*. GA is shown for comparison with permission from Dr. Philippe Brûlet. **b** Reconstitution with CLZ-*f*. **c** Ca^2+^ titration of Redquorin reconstituted with CLZ-*native*, CLZ-*f*, and CLZ-*hcp. Dashed vertical lines* are drawn at pCa 6

For a detailed characterization of CitA, tdTA, and Redquorin, we examined the relationship between the fractional rate of luminescence (*L*/*Lmax*) and Ca^2+^ concentration by in vitro calibration (Fig. 3). Recombinant proteins were produced in bacteria, affinity-purified, and reconstituted with CLZ-*native*, CLZ-*f*, or CLZ-*hcp*.

The calibration curve of *native*-CitA was very similar to that reported for GA (Fig. 3a), with an EC_50_ of 0.5 μM (Table 1). In contrast, tdTA and Redquorin displayed lower Ca^2+^ sensitivity and less counts than CitA at pCa 6 (Fig. 3a and Table 1). Similar relative differences were found when the proteins were reconstituted with CLZ-*f* (Fig. 3b, Table 1), which provided about an order of magnitude more fractional rate of light emission (*L*/*Lmax*) than CLZ-*native* for all variants, in agreement with previous data on wt Aeq [28]. The sensitivity of tdTA and Redquorin was similar, except for a deviation around pCa 7 (Fig. 3a, b). Figure 3c shows the marked effect of CLZ on Ca^2+^ sensitivity of Redquorin (*hcp* > *f* > *native*).

**Table 1:** Sensitivity of FP-Aeq variants derived from best-fit curve of in vitro calibration data

	CLZ-*native*					CLZ-*f*			CLZ-*hcp*
	Aeq [21]	CitA	GA [4]	tdTA	Redquorin	CitA	tdTA	Redquorin	Redquorin
EC_50_ (μM)	1.27	0.56	0.64	1.13	1.53	0.26	0.61	0.79	0.31
Relative intensity at pCa 6	1.0	39.4	17.8	1.6	1.0	465.7	19.3	14.8	335.0
Log (*L*/*Lmax*) at pCa 6	−3.44	−1.85	−2.19	−3.24	−3.43	−0.78	−2.16	−2.27	−0.92

**Fig. 4:**
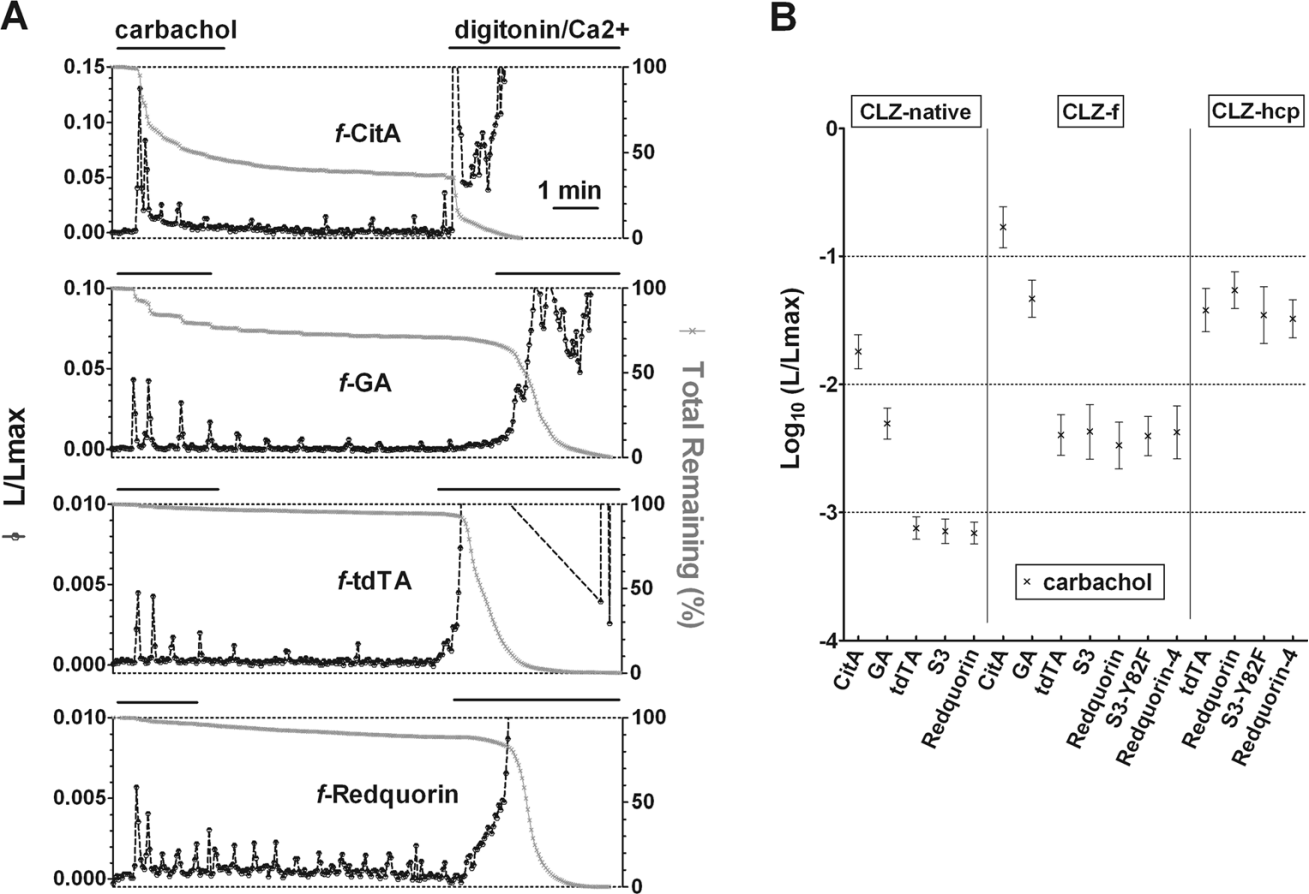
Ca^2+^ sensitivity of FP-Aeq variants obtained in live cells. **a** Time course of bioluminescence of HEK-293 cells expressing the chimeras. Cells were incubated with CLZ-*f*, placed on the microscope stage, and stimulated with carbachol (50 μM) and later permeabilized with digitonin (30 μM) in the presence of Ca^2+^ to release all remaining counts (representative experiments). The *left Y*-axis shows the fractional rate of luminescence (*L*/*Lmax*) and the *right Y*-axis shows the percentage of counts remaining as a function of time. Different *L*/*Lmax* scales were used to reveal the Ca^2+^ oscillations, which prevented showing the full digitonin response. Images were acquired at 3 s per frame, and the intensity was averaged in single cells. **b** Cells expressing the indicated chimeric protein were incubated with CLZ-*native*, CLZ-*f*, or CLZ-*hcp*, exposed to carbachol and later permeabilized with digitonin/Ca^2+^, as shown in **a**. Log (*L*/*Lmax*) values correspond to the peak of the first Ca^2+^ response obtained with carbachol. The average of 11–42 cells from three to six independent experiments is shown for each variant

We also devised a protocol for estimating the Ca^2+^ sensitivity of the chimeras in live mammalian cells: a stimulus raising cytosolic Ca^2+^ to about 1 μM was followed by Ca^2+^ saturation of the probe; the fractional light intensity at 1 μM Ca^2+^ was an indication of the sensitivity of the probe. HEK-293 cells expressing the chimeras were reconstituted with CLZ-*f*, imaged, and stimulated with the muscarinic receptor agonist carbachol, followed by release of all remaining counts with digitonin and high Ca^2+^. Then, the fractional rate of luminescence (*L*/*Lmax*), which is proportional to Ca^2+^ concentration, was calculated in each cell over time (Fig. 4a).

Ca^2+^ oscillations were observed with the green- and red-emitting Aeq variants on addition of carbachol (Fig. 4a). Based on earlier calibration with synthetic indicators, Ca^2+^ levels in HEK-293 cells stimulated with carbachol were estimated to rise to about 1 μM [31] and reached saturating Ca^2+^ by incubation with digitonin/Ca^2+^. In the representative single cells of Fig. 4a, CitA and GA showed larger *L*/*Lmax* at the first Ca^2+^ spike (and faster consumption rate, right *Y*-axis) than tdTA or Redquorin, in agreement with the in vitro calibration shown before.

Using this live-cell protocol, the Ca^2+^ sensitivity of the variants of Fig. 1 was estimated for CLZ-*native*, CLZ-*f*, and CLZ-*hcp* (Fig. 4b). tdTA, Redquorin, Redquorin-4, and variants S3 and S3-Y82F had similar Ca^2+^ sensitivity for a given cofactor but were less sensitive than CitA or GA. Remarkably, the fractional light emission (*L*/*Lmax*) obtained in cells closely matched that obtained in vitro (cf. Table 1 and Fig. 4b). Figure 4b also shows the effect of CLZ on Ca^2+^ sensitivity for all FP-Aeq variants.

### Imaging spontaneous Ca^2+^ activity in HEK-293 cells using CLZ-*hcp*

**Fig. 5:**
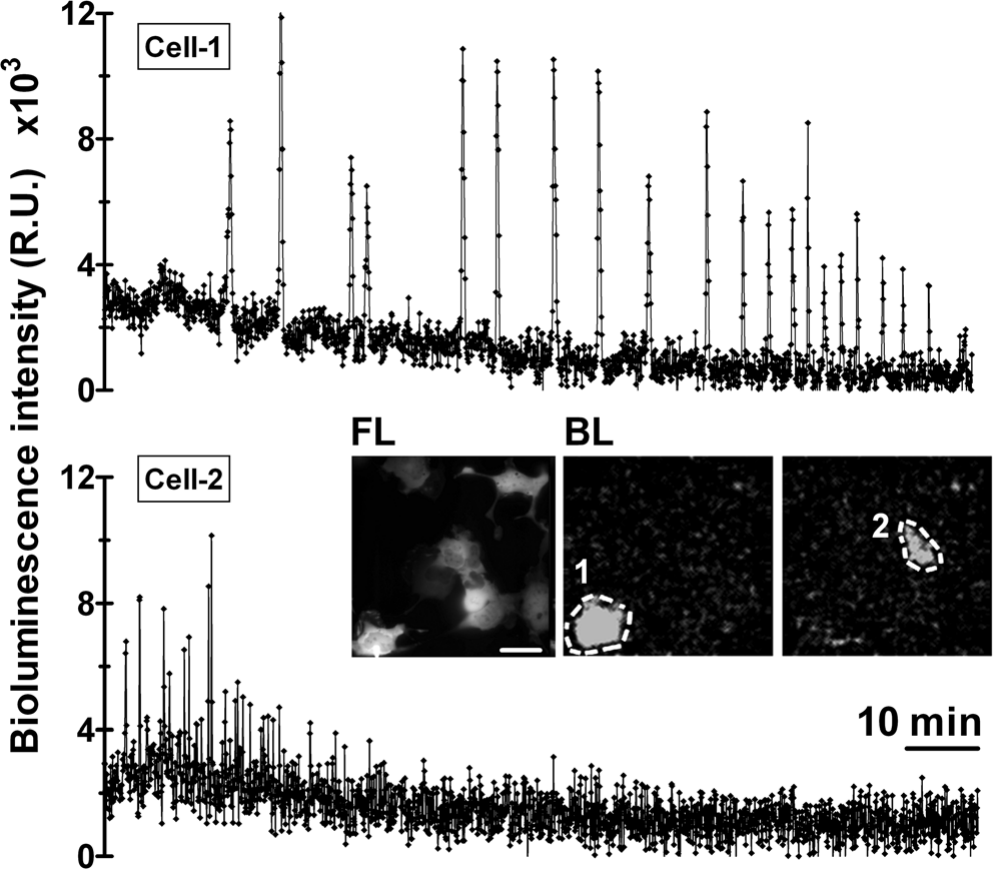
Spontaneous Ca^2+^ oscillations in individual HEK-293 cells visualized with *hcp*-Redquorin. Cells were imaged with controlled humidity at 37 °C and 5 % CO_2_ on the microscope stage. The *inset* shows fluorescence (*FL*) and bioluminescence images (*BL*) of two cells corresponding to the traces shown. *Scale bar*, 20 μm. Integration time was 4 s/frame (representative experiment out of three)

The emission spectrum, emission rate, sensitivity toward Ca^2+^, and reaction kinetics of Aeq change with the CLZ employed for reconstitution of the apoprotein. Thus, to match the Ca^2+^ levels of cell compartments or microdomains of high [Ca^2+^], Aeq Ca^2+^ affinity can be decreased by using CLZ-*n*. In addition, it can be further reduced by mutations in the Aeq Ca^2+^ binding sites [12, 17, 18]. On the other hand, some cells are known to have very low cytosolic [Ca^2+^] at rest, less than 100 nM [25]. To study such systems, indicators with high Ca^2+^ affinity should be used [11]. When combined with wt Aeq, CLZ-*hcp* has been shown to provide the highest emission rate (190-fold more quanta/s than CLZ-*native* at pCa 7) and fastest kinetics (half-rise 2–4 ms and half-decay 150–300 ms) [27], which are good properties for detecting minute changes occurring during spontaneous Ca^2+^ oscillations. Our titration data (Figs. 3c and 4b and Table 1) suggests that *hcp*-Redquorin may provide sufficient sensitivity to measure these low Ca^2+^ signals. In fact, in unstimulated HEK-293 cells, *hcp*-Redquorin allowed imaging spontaneous Ca^2+^ oscillations continuously for up to 5 h (Fig. 5). Thus, Redquorin is able to detect a broad range of Ca^2+^ levels in single mammalian cells, a demanding application because of the limited light output. It reports agonist-induced cytoplasmic Ca^2+^ oscillations and also functions as an ultrasensitive indicator, even using non-photon counting detectors (an EM-CCD).

### Expression of Redquorin and in vivo luminescence in zebrafish embryos

**Fig. 6:**
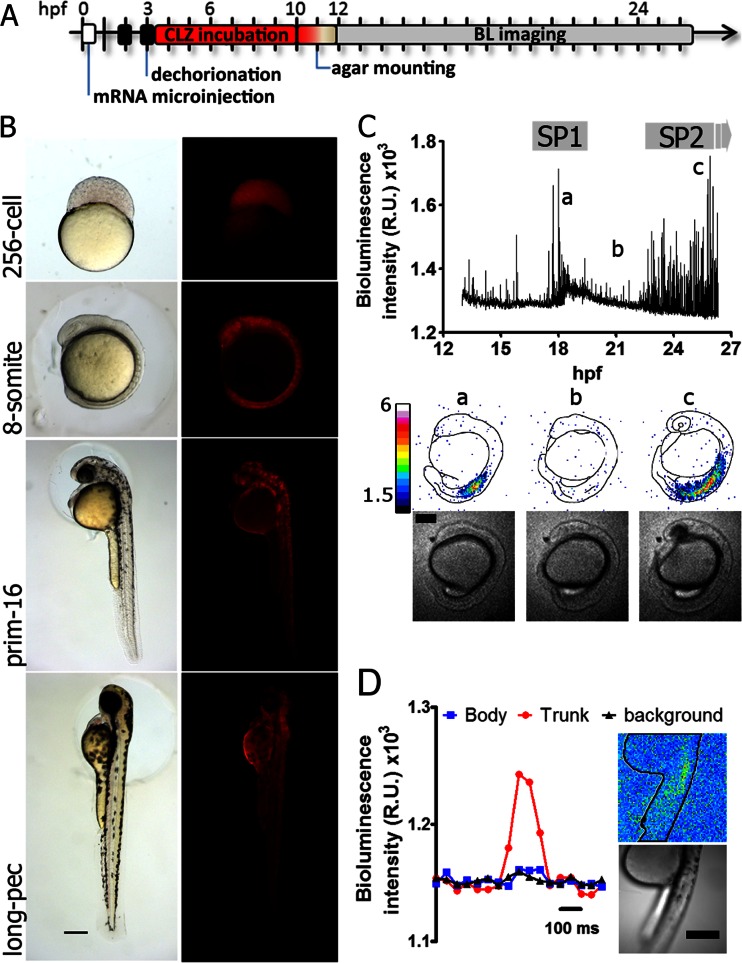
Imaging spontaneous Ca^2+^ oscillations in Redquorin-expressing zebrafish. **a** Steps of a typical experiment include mRNA microinjection, reconstitution with CLZ-*f*, and imaging (hours post-fertilization (hpf)). **b** Transmitted light and red fluorescence images of embryos expressing Redquorin during early development. **c** Bioluminescence time course showing spontaneous Ca^2+^ signals (10 s/frame, whole body field). Selected bioluminescence (relative units ×1,000) and transmitted images are shown. **d** A single Ca^2+^ transient in the trunk in the SP2 period, imaged with fast acquisition (integration time was 0.05 s/frame). Average counts in regions of interest on the body and outside of the specimen (*background*) are shown. *Scale bars*, 250 μm

Having demonstrated the use of Redquorin in single cells, we then tested the biosensor in vivo. Zebrafish embryos have been shown to be outstanding specimens for imaging in early developmental stages. In zebrafish embryos at one-cell stage, mRNA coding for Redquorin was injected in the center of the yolk or close to the blastodisc. Red fluorescence was detected as early as 2.5 hpf (256-cell stage) and up to 53 hpf (long-pec stage) (Fig. 6a, b). Redquorin showed ubiquitous mosaic expression as seen by fluorescence and most injected embryos developed normally.

Bioluminescence could be detected as soon as 3 hpf when dechorionation for cofactor reconstitution was done at 2 hpf. We were able to record Ca^2+^ signals up to 32 hpf using a light-tight microscope and an EM-CCD camera. Since the probe was not targeted in transiently expressing embryos, the signals came from undefined cells.

In long-term experiments, bioluminescent signals were imaged continuously from 12 to 30 hpf (with 1-min or 10-s exposure) using *f*-Redquorin. Spontaneous Ca^2+^ peaks were mainly localized in the trunk region (Fig. 6c and Supplementary video 1), presumably corresponding to the SP1 and SP2 signaling periods in slow muscle cells observed by Miller and coworkers [6]. These authors proposed that the SP1 period was mediated by neuronal activation and was involved in myofibril organization, whereas SP2 correlated with spontaneous side-to-side myotomal contractions. Fast image acquisition (20 fps) was also achieved during spontaneous tail contractions at 30 hpf in partially restrained embryos with free tail, showing the rise and decay of the Ca^2+^ signal during a single twitch (Fig. 6d).

Although the previous experiments were performed without emission filters, Ca^2+^ signals could be seen through a 560–600-nm bandpass filter, but not when using a 470–490-nm filter (Supplementary Fig. 3), in agreement with the red emission of Redquorin.

## Discussion

In this work, we have improved the efficiency of BRET between donor Aeq and acceptor tdTomato in our previously described construct, tdTA [1]. In the red-shifted variant Redquorin, we shortened the original linker and deleted some residues on the C terminus of tdTomato and the N-terminus of Aeq. An intriguing question is why high energy transfer between Aeq and GFP (or citrine) was obtained with a variety of linkers of different length [2], whereas to obtain a similar effect with tdTomato (Fig. 2a), we had to use very short linkers to decrease donor-acceptor distance (Fig. 1). *Aequorea*-derived GFP has coevolved with Aeq, with which it seems to form a complex in the presence of Ca^2+^ [10]. It is likely that this property is shared by mutagenic variants of *Aequorea* GFP (citrine, Venus), but not by FP from other origin, such as *Discosoma*-derived tdTomato.

Another way to maximize BRET from Aeq to tdTomato was to increase the overlap between donor emission and acceptor absorption. Thus, it was useful to red-shift Aeq emission using mutation Y82F (variants S3-Y82F and Redquorin-4), as expected from the literature [30]. Aeq peak emission may also be tuned by choice of the CLZ cofactor. We chose CLZ-*f* for most experiments because it causes wt Aeq to emit at longer wavelengths than other CLZ (473, 464, and 444 nm for CLZ-*f*, CLZ-*h* and CLZ-*hcp*, respectively) [27]. Accordingly, CLZ-*f* resulted in the highest energy transfer of the three CLZ tested with Redquorin (Supplementary Table 1).

The Ca^2+^ sensitivity of an Aeq-based sensor also determines its emission and consumption rates. CLZ-*f* provided an intermediate Ca^2+^ sensitivity compared with CLZ-*native* or CLZ-*hcp* (Figs. 3 and 4b; Table 1). GA and CitA reconstituted with CLZ-*f* were rapidly consumed (see total remaining Aeq in Fig. 4a). In contrast, *f*-Redquorin could be used in longer experiments, providing the camera can detect the lower signals, and allowed Ca^2+^ imaging for hours in early stages of development in zebrafish.

Ultrasensitive Ca^2+^ indicators, YC-Nanos (with *K*_*d*_ values from 15 to 140 nM), allowed detection of subtle Ca^2+^ transients accompanied with spontaneous cell network activity [11]. In this work, we have demonstrated that *hcp*-Redquorin displays high Ca^2+^ sensitivity (EC_50_ 310 nM) (Fig. 3c) and can detect spontaneous Ca^2+^ oscillations in mammalian cells (Fig. 5). To detect fast spontaneous twitching in zebrafish, with hundreds of cells expressing the chimera, less sensitive *f*-Redquorin was used instead (Fig. 6d).

An important point to address is the advantages and limitations related to the use of Aeq probes compared to synthetic or gene-encoded fluorescent Ca^2+^ indicators. Regarding the time resolution achievable with bioluminescence, it is limited by Aeq kinetics and by the low photon yield of the photoprotein. Although the half-rise time of Aeq upon Ca^2+^ binding has been found to be from 2 to 30 ms, decay times are slower [27]. In addition, since Aeq emits one photon per molecule (or none), light output is much less than in fluorescence. This is compensated by the low background characteristic of bioluminescence, which results in a high signal-to-noise. Thus, the photoprotein can be used at two to three orders of magnitude lower concentration than synthetic Ca^2+^ indicators [21]. For the above reasons, imaging single cells with Aeq is more demanding than doing luminometry in cell populations or using fluorescence probes. Modern EM-CCD cameras are sensitive enough for this use and can replace photon counting detectors [22]. To detect Redquorin with our imaging device, an EM-CCD, we routinely used 1–3 s per image frame, which was sufficient to resolve cytoplasmic Ca^2+^ oscillations in HEK-293 cells (Figs. 4 and 5). The practical limit was 350 ms/frame for detecting Ca^2+^ rises in response to carbachol. Many fluorescent indicators achieve better time resolution and are able to resolve fast Ca^2+^ influx in single excitable cells. However, continuous imaging would cause phototoxicity and photobleaching of the fluorescent probe. This shortcoming is completely absent with Aeq probes and experiments of several-hour duration with continuous image acquisition are feasible. Nowadays, a large array of genetic techniques is available to target Aeq probes and gene-encoded fluorescent Ca^2+^ indicators (GECI) to particular tissues or subcellular locations. However, while Aeq is entirely exogenous to expression systems, calmodulin present in some of these GECI can interfere with the endogenous signaling.

Luminescent and fluorescent probes are thus complementary and the amplitude, kinetics, duration of the Ca^2+^ response, as well as location (subcellular, cell, organism, depth of tissue) should guide the choice of method. In short, fluorescent probes are best for imaging fast events in single cells; for long experiments with minimal toxicity, Aeq probes are unsurpassed. In depth, reviews provide a more detailed appraisal of the relative advantages and drawbacks of Aeq versus fluorescent Ca^2+^ probes [3, 16, 20, 21, 33].

When dealing with experiments in cell populations in vitro or in vivo, Aeq-based probes offer several other benefits. For example, we showed that myotomal contractions were easily detected in zebrafish imaged continuously for up to 18 h, during which time development proceeded normally. In this sort of specimen, Ca^2+^ fluctuations have variable spatiotemporal properties, which would be hard to reveal with a fluorescent probe for so long as reported here. Since the Ca^2+^ rises were due to synchronous muscle contractions, the time resolution could be increased up to 20 fps (0.05 s/frame) (Fig. 6d). Thus, low photon yield was compensated with a large number of emitting cells to form an image with high signal-to-noise. Moreover, the imaging setup is simple, since no excitation equipment is needed. For the same reason, studies in free-moving animals are facilitated [23] and problems associated with poor tissue penetration of excitation light or phototoxicity are also avoided. However, loading of CLZ may constitute an important drawback, particularly in some tissues in mammalian models (e.g., across the brain–blood barrier); simple incubation with CLZ sufficed in zebrafish embryos.

Imaging in live organisms introduces a noteworthy problem associated to light scattering and absorbance of tissues (mainly blood and bone) which lead to a significant loss of photons. Unlike earlier FP-Aeq chimeras, Redquorin’s red emission component was efficiently transmitted in absorbing tissues such as blood, and therefore, Redquorin has the potential to allow Ca^2+^ imaging in deep tissues of living organisms. To this end, transgenic organisms expressing Redquorin and other Aeq probes should be generated. Ca^2+^ sensitivity could be tuned without the need for creating new transgenic lines by using different CLZ (Figs. 3 and 5).

In conclusion, Redquorin is the Aeq-based Ca^2+^ sensor with the strongest red emission reported thus far. It can be combined with CLZ analogs to fine-tune properties such as Ca^2+^ affinity and emission peak. In addition to detecting oscillations elicited by Ca^2+^-mobilizing agents, this biosensor allowed imaging spontaneous Ca^2+^ activity in mammalian cells, as well as during zebrafish development. Redquorin’s emission profile will be useful for intravital Ca^2+^ imaging in mammals and to perform simultaneous dual-color imaging in different cells or compartments by combining it with blue-green Aeq probes [15].

## Electronic supplementary material

Below is the link to the electronic supplementary material.

ESM 1 (PDF 729 kb)

ESM 2 (AVI 2350 kb)
