# Supplementary material for: Imaging Ca2+ activity in mammalian cells and zebrafish with a novel red-emitting aequorin variant
Source: Pflugers Arch. 2014 Oct 31;467(9):2031–42. doi: 10.1007/s00424-014-1639-3 (PMC4537489; doi:10.1007/s00424-014-1639-3)
Supplement: Supplementary file 1 — (PDF 729 kb) [file 424_2014_1639_MOESM1_ESM.pdf]

Imaging Ca^{2+} activity in mammalian cells and zebrafish with a novel red-emitting aequorin variant

Adil Bakayan[a], *Beatriz Domingo*[a], *Atsushi Miyawaki*[b] *and Juan Llopis*[a]

[a]Centro Regional de Investigaciones Biomédicas (CRIB) and Facultad de Medicina de Albacete, Universidad Castilla-La Mancha, Spain

[b]Laboratory for Cell Function Dynamics, Brain Science Institute, RIKEN, Hirosawa, Saitama 351-0198, Japan

Figure S-1: Subcellular localization of fluorescence of tdTA variants expressed in HeLa cells.

Figure S-2: Western blot of tdTA, Redquorin and CitA.

Figure S-3: Luminescence from zebrafish expressing Redquorin into red and blue emission channels.

Table S-1: Characterization of tdTA variants using the four-channel approach.

Table S-2: Oligonucleotides used for the construction of variants with different linkers and point-mutations.

Video S-1. Spontaneous Ca^{2+} transients of an *f*-Redquorin expressing zebrafish embryo.

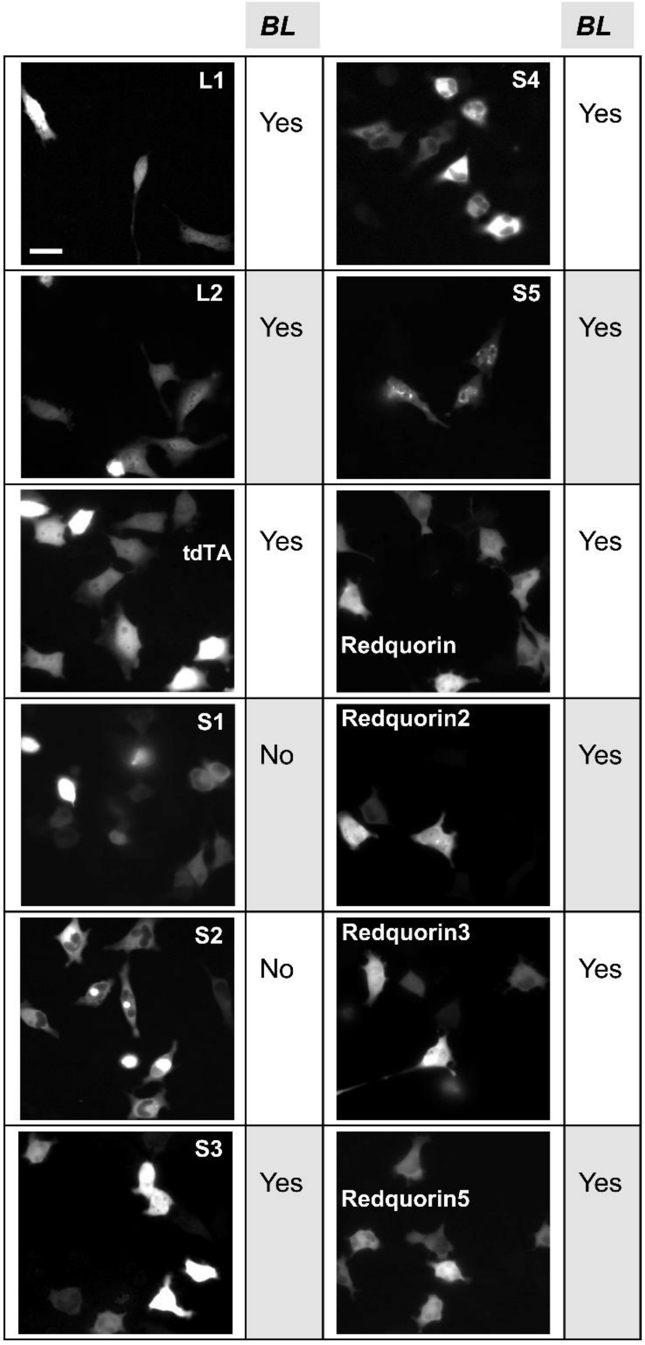

Figure S-1. Subcellular localization of fluorescence of tdTA variants expressed in HeLa cells. Representative fluorescence images of each variant are displayed. The presence or absence of bioluminescence (BL) is indicated. Images were taken with 40x objective. Scale bar is 30 μm.

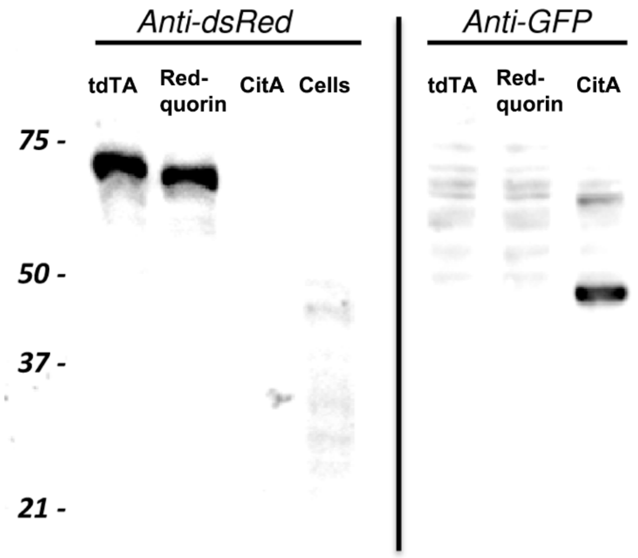

Figure S-2. Western blot of tdTA, Redquorin and CitA. Lysates of untransfected HeLa cells, or cells expressing tdTA, Redquorin or CitA were run in SDS gels, transferred to nitrocellulose and probed with anti-dsRed (left) or anti-GFP (right). Markers of molecular weight (kDa) are displayed on the left.

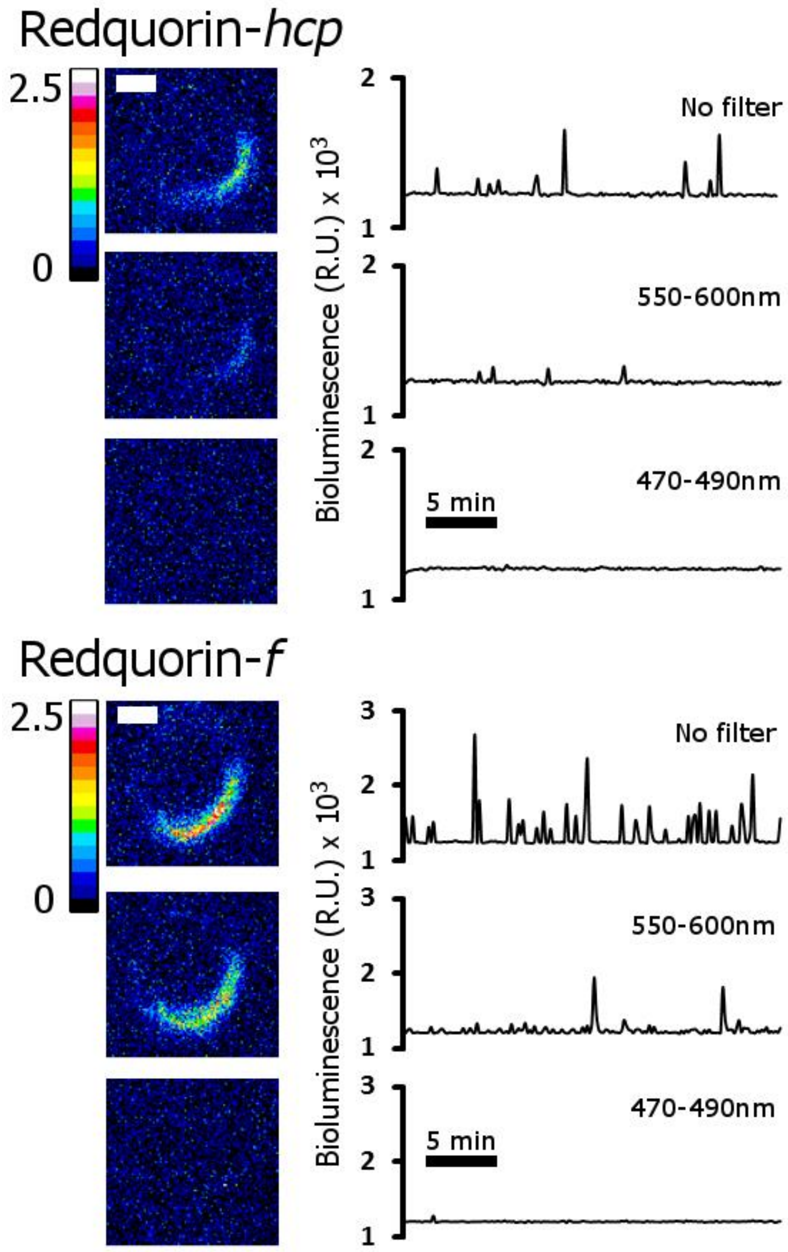

Figure S-3. Luminescence from zebrafish expressing Redquorin into red and blue emission channels. No emission filter, 560-600 nm, or 470-490 nm bandpass emission filters were used. Images obtained during Ca^{2+} transients and the luminescence time-course at 32 hpf are shown. ApoAeq was reconstituted with CLZ-*hcp* or -*f*. Scale bar, 250 μm.

Table S-1. Characterization of tdTA variants using the four-channel approach.

		Bioluminescence contribution (% of total)					
**CLZ analog**	**tdTA variant**	**481 nm (B)**	**535 nm (G)**	**595 nm (O)**	**640 nm (R)**	**B+G**	**O+R**
f	Aeq	60.2 ± 2.1	29.2 ± 1.3	7.2 ± 1.9	3.4 ± 1.0	89.4	10.6
	GA	16.2 ± 4.1	72.3 ± 2.5	9.4 ± 4.0	2.1 ± 1.1	88.5	11.5
	tdTA	38.1 ± 2.5	20.0 ± 2.1	28.6 ± 2.6	13.3 ± 1.5	58.1	41.9
	tdTA-Y82F[b]	25.0 ± 3.2	26.0 ± 2.8	33.0 ± 4.0	16.0 ± 2.2	51.0	49.0
	L1	44.6 ± 4.1	22.4 ± 2.2	22.3 ± 3.6	10.7 ± 2.7	67.0	33.0
	L2	41.3 ± 5.1	21.1 ± 3.6	25.7 ± 4.0	11.9 ± 3.0	62.4	37.6
	S3	33.0 ± 3.2	18.3 ± 1.6	32.3 ± 2.9	16.4 ± 1.6	51.3	48.7
	S3-Y82F[b]	20.8 ± 3.5	20.5 ± 2.1	39.1 ± 4.1	19.6 ± 2.0	41.3	58.7
	S4	48.6 ± 3.5	25.8 ± 1.7	17.4 ± 3.0	8.2 ± 1.5	74.4	25.6
	S5	33.3 ± 2.2	19.0 ± 1.6	32.2 ± 1.4	15.5 ± 1.9	52.3	47.7
	Redquorin-2	26.0 ± 2.6	9.0 ± 1.2	45.7 ± 2.1	19.3 ± 1.8	35.0	65.0
	Redquorin-3	13.8 ± 3.6	11.7 ± 1.7	50.0 ± 1.0	24.5 ± 3.6	25.5	74.5
	Redquorin	12.6 ± 2.4	10.0 ± 1.6	50.8 ± 3.2	26.6 ± 1.3	22.6	77.4
	Redquorin-4[b]	7.6 ± 1.1	10.4 ± 1.3	54.0 ± 1.4	28.0 ± 1.1	18.0	82.0
	Redquorin-5[b]	15.3 ± 2.4	18.7 ± 2.8	43.9 ± 3.0	22.1 ± 2.2	34.0	66.0
hcp	tdTA[a]	51.5 ± 2.5	17.0 ± 1.1	20.7 ± 2.6	10.8 ± 0.7	68.5	31.5
	tdTA	52.0 ± 2.7	16.8 ± 0.9	20.8 ± 2.1	10.4 ± 1.2	68.8	31.2
	S3-Y82F[b]	29.9 ± 3.9	19.1 ± 2.5	33.5 ± 4.0	17.5 ± 2.4	49.0	51.0
	Redquorin[a]	15.0 ± 2.2	10.3 ± 1.3	50.1 ± 2.5	24.6 ± 1.2	25.3	74.7
	Redquorin	18.0 ± 3.9	9.6 ± 1.5	47.6 ± 3.5	24.8 ± 1.7	27.6	72.4
	Redquorin-4[b]	16.5 ± 2.1	10.9 ± 1.0	45.7 ± 1.1	26.9 ± 1.3	27.4	72.6
h	tdTA	41.5 ± 2.4	18.9 ± 1.9	27.5 ± 2.2	12.1 ± 2.5	60.9	39.6
	tdTA-Y82F[b]	28.6 ± 3.3	25.7 ± 3.0	31.8 ± 3.9	14.0 ± 2.2	54.3	45.8
	L2	44.5 ± 1.3	20.2 ± 2.2	24.6 ± 2.6	10.7 ± 1.6	64.7	35.3
	L1	48.6 ± 1.9	22.1 ± 1.6	20.8 ± 2.1	8.5 ± 1.8	70.7	29.3
	Redquorin	13.3 ± 3.2	12.6 ± 1.8	48.4 ± 3.5	25.7 ± 2.0	25.9	74.1
	Redquorin-4[b]	10.8 ± 3.3	10.9 ± 0.8	51.7 ± 3.4	26.6 ± 0.7	21.7	78.3
	Redquorin-5[b]	18.2 ± 2.0	17.8 ± 2.9	43.1 ± 2.9	20.9 ± 1.8	36.0	64.0

[a] Data were obtained in HEK cells stimulated with 50 μM carbachol instead of digitonin.
[b] These clones contain Aeq point-mutation Y82F.

HeLa cells expressing Aequorin (Aeq), GFP-Aeq (GA), tdTA, and its variants (Fig. 1) were reconstituted with the indicated CLZs. The percentage of counts in four emission channels (center wavelength is indicated) were determined in individual HeLa cells after permeabilization with digitonin/Ca^{2+} (see[1]). The average of 6 to 38 cells ± S.D. is shown. The two rightmost columns show the sum of % emission in the 481 and 535 nm channels (B+G, blue plus green), and the sum of 595 and 640 nm channels (O+R, orange plus red).

Table S-2. Oligonucleotides used for the construction of variants with different linkers and point-mutations.

Name	Oligonucleotide DNA sequence
22aa-L	5′- CCGGGGTGGCAGTGGAAGTGGTCAAAGTGGTTCCGGTAGTGGAGGCCAGAGTGGTAGTGGAAGTGGCCAATCC<br>3′- CACCGTCACCTTCACCAGTTTCACCAAGGCCATCACCTCCGGTCTCACCATCACCTTCACCGGTTAGGGGCC
13aa-L	5′- CCGGGGTAGTGGAGGCCAGAGTGGTAGTGGAAGTGGCCAATCC<br>3′- CCATCACCTCCGGTCTCACCATCACCTTCACCGGTTAGGGGCC
6aa-L	5′- CCGGCGGCAGCGGTAGCT<br>3′- GCCGTCGCCATCGAGGCC
YG-SG	(P)5′- CTGTTCCTGTCCGGAATGGACGAGCTGTAC
LF-SG	(P)5′- CCGCCACCACTTGGGCCTGTACGGCATGGACG (1st round)<br>(P)5′- CCGCCACCACTCCGGACTGTACGGCATGGACG (2nd round)
DN-SG	(P)5′-CTTACATCAGACTTCGCCAGACCAAGATGGATTGGACG (1st round)<br>(P)5′- TACATCAGACTTCTCCGGACCAAGATGGATTGGAC(2ndround)
SD-SG	(P)5′- GATCTGTCAAACTTACATCCGGATTCGACAACCCAAGATG
SV-SG	(P)5′- CAGTCCGGACTCAGATCCGGAAAACTTACATCAGACTTCG
G-D	(P)5′- CCACCTGTTCCTGTCCGACTTCGACAACCCAAGATGG
F224L	(P)5′- GAGGGCCGCCACCACCTGCTGCTGTCCGGATTCGACAAC
Y82F	(P)5'- GTGGAAACTGATTGGCCTGCATTTATTGAAGGATGGAAAAAATTG

The table is divided in three sections by dashed lines. Oligonucleotides used to construct long-linker peptide variants are shown in the top section. The middle section lists the mutagenic oligonucleotides used to convert aminoacids in tdTA to SG, D or L, as indicated in Fig. 1 and in Materials and methods. Some aminoacid changes required two

rounds of mutagenesis. The last section displays the oligonucleotide used to insert Y82F mutation in Aeq. Mutagenic oligonucleotides carried a phosphate group (P) at their 5'-end. All oligonucleotides were synthesized by Fisher Scientific.

Video S-1. Spontaneous Ca^{2+} transients of an *f*-Redquorin expressing zebrafish embryo at 26 hpf. Brightfield and luminescence timelapse images are shown. Integration time was 10 s/frame. Time stamp indicates hours:min post-fertilization. Scale bar, 250 µm. Luminescence is shown in relative units x 1000.
